# Anatomico-Pathological Remarks on the Inflammation of Some Internal Parts of the Eye, and Especially on Choroiditis, as the Cause of Glaucoma

**Published:** 1844-07

**Authors:** 


### ART. III.

*Anatomisch Pathologische Opmerkingen over de Ontsteking van eenige Inwendige Deelen van het Oog, en bijzonder over Choroiditis als Oorzaak van Glaucoma.* Door J. L. C. SCHROEDER VAN DER KOLK, Hoogleeraar in de Geneeskunde te Utrecht. (*No Date.*)

*Anatomico-pathological Remarks on the Inflammation of some internal parts of the Eye, and especially on Choroiditis, as the Cause of Glaucoma.* By J. L. C. SCHROEDER VAN DER KOLK, Professor of Medicine at Utrecht. 4to, pp. 26. One Coloured Plate.

THIS memoir was published in the first number of the Transactions (1841) of the Society at Amsterdam for the Promotion of Medicine and Surgery. Independently of the high character borne by the author as a pathologist, the paper itself would justly claim our attention. It is divided into two parts, the first anatomical, and the second pathological.

The author commences by some remarks on the importance of pathological anatomy. If this science, he observes, is indeed to be fruitful, and to afford any explanation of the origin, cause, and course of different diseases, it must by no means be confined to recitals merely of the morbid changes presented by single parts of the body, nor to delineations and descriptions of the form and colours of certain morbid degenerations, but it must go hand in hand with an accurate knowledge of the structure and functions of the various organs in the healthy state. However much has been done by descriptions and engravings to elucidate the diseases of the eye, he thinks that for want of sufficiently exact anatomical and pathological observations, there is yet much uncertainty, and even confusion, in the knowledge hitherto acquired of the origin and nature of many of the diseases of that important organ. To judge of its diseases, and distinguish them with accuracy, it would be necessary to possess an exact knowledge of the system of the eye in general, and especially of the course and distribution of its nerves and finer vessels; upon which latter point there reigns, according to the Professor, yet great uncertainty.

In respect to inflammations especially, it is of great importance to distinguish those which are external from those which are internal; a matter not always easy, because the internal inflammations of the eye, above all if they follow a chronic course, are not always even visible during life. In order to understand this subject fully, it is necessary to be aware that the vessels which are distributed externally to the conjunctiva, are by no means directly connected with the internal vessels of the eye, so that a severe external inflammation or degeneration may exist for a long time without communicating any diseases to the internal parts of the organ. The internal nerves, *nervi ciliares*, seem still less connected with the external ones, which give sensibility to the conjunctiva. The latter arise principally from the lacrymal nerve, which holds no immediate connexion with the branches of the ophthalmic ganglion; a wise precaution, our author remarks, of nature. He thinks it doubtful whether the branches of the ciliary nerves, which, according to Schlemm and Valentin, pierce the edge of the cornea, are connected with the nerves of the conjunctiva. At all events, the connexion can be of very trifling influence, as appears from the frequent and severe external affections of the eye, which occur without any morbid change of the internal parts. In illustration of this,

LIBRARY OF THE

Orleans Parish Medical Society

Dr. Schroeder van der Kolk mentions a case of melanosis, in which the eye was extirpated, and in which he succeeded, notwithstanding the necessary division of many of the vessels, in making a very successful injection, even of the finest branches. All the internal parts of the eye appeared quite healthy, although the fungous growth from the sclerotica and cornea was half an inch, and in some places, a full inch in thickness. The same fact derives additional confirmation from the results of the operation for strabismus; the division of the conjunctiva, and of one or several of the muscles of the eyeball, never causing any internal ophthalmia.

Contemplated in a pathological view, there is, among the internal parts of the eye, none, according to Dr. Van der Kolk, except the retina, more important than the choroid. He says, it is that part of the eye in which by far the greatest number of blood-vessels and nerves of the vegetative life are united, and which possesses in a high degree all the conditions fitting it for inflammation. The choroid, besides, gives vessels to almost all the internal parts of the eye, so that an attack originating there may give rise to the most diversified effects. As the state of the choroid cannot be fully investigated except in the dead body, its inflammation is in many manuals not noticed at all; and although some observations and separate treatises have appeared on choroiditis, Dr. Van der Kolk considers the importance of its attacks, and its manifold existence in internal disorders of the eye, to have met with by far too little attention. If the disease becomes chronic, which often happens, it is still more easily mistaken, so that it is almost always the acute form which is described. Our author proceeds to explain the fine connexions of the vessels of the choroid with those of the other parts of the eye, before taking up the anatomical consideration of some of the diseases of this organ.

The choroid, he remarks, is nothing else than a continuation of the pia mater of the brain. Externally, it is covered by the vorticose veins, while on its internal surface, otherwise called the *membrana ruyschiana*, the fine arterial ramifications are found which secrete the pigment. These vessels, however, do not end in the choroid, but pass over into the iris. By means of a beautiful distribution, they form the *corona ciliaris* or ciliary processes. The arched folds of which these processes consist are received between the folds of the *zonula zinnii*, which surrounds the lens, and forms the anterior part of the hyaloid membrane. The connexion between the two zones is rendered much more intimate, Dr. Van der Kolk observes, from very many of the finest capillaries passing out of the fringes of the *corona ciliaris* into the *zonula zinnii*, whence arise ramifications of the highest importance, which have not been described with sufficient exactness. This transition of vessels from the choroid to the hyaloid zone has been represented by several anatomists as seen in the eye of the fœtus born before the full time, but it has been doubted whether it could be seen in the full-grown subject. Dr. Van der Kolk has, by a lucky injection, filled the vessels in question in the healthy adult eye, and has observed extremely fine vessels passing from the exterior circumference of the zonula zinnii into the hyaloid membrane.

The vitreous humour, according to our author, is surrounded by a very thin serous membrane, through which run some extremely minute vessels, which are rarely injected. If we take off this membrane from the vitreous humour, which once happened to our author in an excellently injected eye, it is then evident that the very delicate vessels in question spread along the membrane. On account of its perfect transparency and extreme tenuity, it is indeed scarcely possible to recognize it as a membrane, unless its vessels have been previously filled, which very seldom happens.

These vessels, which probably serve for the secretion of the vitreous humour, arise from two sources. Some shorter ones, which, for distinction's sake, Dr. Van der Kolk calls the *vasa brevia membranæ hyaloideæ,* arise from the exterior margin of the *zonula zinnii,* whence they send their ramifications over the hyaloid membrane, and especially over its anterior part. Other vessels arise from the *arteria centralis retinæ,* just where it commences to spread itself into the retina. Of these vessels, distinguished, by their extreme fineness, and their straighter course, from those of the retina, four seem to be demonstrable. They are called by Dr. Van der Kolk the *vasa longa membranæ hyaloideæ,* and anastomose with the *vasa brevia.* The vessels, then, of the vitreous humour are connected with those of the ciliary processes and the choroid, through the zonula zinnii. The vessels of the retina, which are very abundant, and much firmer and more winding than those of the hyaloid membrane, terminate chiefly in the retina itself, but partly, as appears from a preparation in the possession of Dr. Van der Kolk, they unite with the fine vascular band of the zonula zinnii and ciliary processes.

From all this it appears, that the vessels of the choroid are connected with the iris, corona ciliaris, zonula zinnii, retina, and corpus hyaloideum. From this general union arise some still finer vessels. In the very successfully injected eye already referred to, some extremely fine ramifications pass from the interior margin of the zonula zinnii and corona ciliaris to the anterior surface of the capsule of the lens. The branch of the arteria centralis retinæ, which is demonstrable in the fœtus, piercing the vitreous humour, to reach the posterior surface of the capsule of the lens, vanishes completely in later life. Dr. Van der Kolk suspects that in the adult some extremely fine branches pass from the zonula zinnii, and especially from the vasa longa membranæ hyaloideæ, towards the posterior surface of the lens. This he gives only as a conjecture, as he has not been able to confirm it by injections.

Besides the vessels above mentioned, there appear to be others, too fine to be artificially filled in a sound eye, which spread from the exterior margin of the iris along the posterior surface of the cornea. In chronic inflammation of the eye, these vessels become so enlarged, that they may be filled with coloured material.

The point, then, whence the greatest number of vessels spread into the internal parts of the eye, and which thus comes to be of the highest importance, is that where the vessels of the choroid and ciliary processes unite with those of the zonula zinnii and retina. It is from this point that branches pass towards the capsule of the lens and hyaloid membrane.

Dr. Van der Kolk goes on to observe that, in all inflammations of the choroid, the ciliary nerves are affected, whereby the motions of the iris become impeded. According to the greater or less affection of some of these nerves, some part of the iris, he says, will be more contracted than

CHARITY HOSPITAL, MEDICAL LIBRARY.

another, an observation with which our readers are, no doubt, already familiar. He also points out the effect which a morbid state of the ciliary nerves must have on the nutrition of the eye. In an atrophic eye he found all the ciliary nerves much more slender than natural, of a transparent bluish colour, and seemingly deprived of their medulla, while the ophthalmic ganglion was much reduced in size. According to Walther and others, affections of the ciliary system are capable of causing blindness. In a remarkable case of ischias which had proceeded to suppuration, and where the ischiatic nerve was imbedded in pus, Dr. Van der Kolk found on dissection the principal veins of the upper part of the head, as the venæ frontales and temporales, and even those of the diploe of the skull filled with pus; also in the cavernous sinus pus was present; the ophthalmic ganglion was red and surrounded by pus. The patient had been phrenitic, and had been affected with violent pain in the head, and the eye had been red and strongly congested; but there was no blindness.

The ciliary system is connected, moreover, Dr. Van der Kolk remarks, with the fifth nerve, whence may be explained the severe pain of the forehead which attends inflammation of the choroid, and which may arise from a reflex effect upon the origin of the frontal nerve.

Our author next proceeds to consider some of the morbid affections of the eye. He begins by observing, that inflammation of the choroid is described, by some authors, under the more general and very indefinite name of *ophthalmitis interna.* The disease, he says, is most distinctly recognized in the acute form. The symptoms are a pressing, distending, dull, but constantly increasing, pain of the whole eye, soon spreading by sympathy to the head; irritation of the retina, constant photopsia, diminution of sight, dimness and contraction of the pupil. There follows discoloration of the iris, which is also pressed towards the cornea; the headach becomes extreme; the conjunctiva and sclerotica redden; the cornea begins to lose its lustre, and at length matter shows itself in the anterior chamber. This description is taken from Weller, who has merely followed Beer. We must confess, we have rarely met with an idiopathic inflammation of the eye, answering to this description. Neither do we see sufficient proof, that the symptoms in question are those of acute choroiditis. They are fully as likely to belong to an acute inflammation of the retina. We consider this part of Dr. Van der Kolk's paper to be defective. He regards the symptoms set down by Beer and Weller as those of ophthalmitis interna to belong to acute inflammation of the choroid, but he cannot surely expect his *ipse dixit* on this point to be acceded to without evidence. Had it been at all manifest that the symptoms in question indicated peculiarly an inflammation of the choroid, Beer would undoubtedly have preferred the name *choroiditis* to *ophthalmitis interna.* Dissections of such cases are wanted, to clear up their real nature.

Dr. Van der Kolk observes, that the chronic form of choroiditis has long been described under an entirely different name. He holds *glaucoma,* in fact, to be the result of chronic inflammation of the choroid. In support of this notion, he insists much that the greenish reflection in glaucoma is of a concave form, whereas did it depend, as the generality of authors have supposed, on an affection of the vitreous homour, it should appear, he thinks, as a sphere. Of the value of this observation, we shall have occasion to say something hereafter. Our author seems altogether unacquainted with the striking proof of the greenish appearance in glaucoma depending entirely on the state of the lens, afforded by the fact, that if the lens is extracted, the greenness and opacity disappear completely, let their apparent form be what it may.

The pupil is dilated and immoveable in glaucoma. This shows, says Dr. Van der Kolk, an affection of the ciliary system, and thus of the choroid, and cannot be explained by an affection of the retina; a remark to which we cannot assent. In many cases of gutta serena, we have a dilated immoveable pupil, without the smallest suspicion of any disease of the choroid. An insensible state of the retina is perfectly sufficient to cause a want of activity in the ciliary nerves. The motion of the pupil is almost entirely a reflex action, depending on the influence of light on the retina. If the retina is rendered by disease incapable of being acted on by light, the reflex effect on the iris is also at an end.

The pupil is not only motionless in glaucoma, says Dr. Van der Kolk, but its shape is changed, being elongated towards the two angles of the eye, as in the ruminantia, showing an unequal action of the ciliary nerves, whereof two are comites of the long ciliary arteries and veins. These two nerves are more affected, he thinks, by the inflammation of the choroid than the others, whence the oval form of the pupil. This form of the pupil has often been described and figured by the German oculists; but it is remarkable, that, although we see the pupil irregularly dilated in glaucoma, we rarely in this country observe the transversely oval pupil.

The loss of sight in glaucoma is not at all proportionate to the change in the colour of the pupil. Hence we may conclude, Dr. Van der Kolk says, that an opacity of the vitreous humour is not the cause of glaucoma. In fact, the deterioration of vision is so great, that all objects appear as if in a cloud, before any change in the pupil is observable, whence some have believed the true cause of glaucoma to be inflammation of the retina. The symptoms of retinitis, however, are very different from those of glaucoma.

The cause of glaucoma, according to Dr. Van der Kolk, resides neither in the vitreous humour nor in the retina, but consists in a chronic inflammation of the choroid, and an effusion of coagulable lymph between that membrane and the retina. This opinion, he tells us, he has adopted, not from any superficial agreement merely between the phenomena of acute or chronic choroiditis, and those of glaucoma, but from anatomical inquiry.

As a continuation of the pia mater, says our author, the choroid belongs to the serous membranes. Now, it is known, that in certain inflammations, acute as well as chronic, an exudation of serum or of coagulable lymph proceeds from such membranes, and this happens, he adds, in chronic choroiditis. He first observed this in the eye of an old woman, which had been ineffectually operated on for cataract by keratonyxis, while the other eye suffered from amaurosis. In the cataractous eye, he found a thick layer of coagulable lymph between the choroid and the retina. Such an effusion, in consequence of choroiditis, completely explains, in his opinion, all the phenomena of glaucoma, which in this woman was combined with cataract. Of the eye in question, Dr. Van der Kolk gives a representation, and we have no doubt a correct one; but the accuracy of his conclusion, that such an effusion is the cause of glaucoma, we can by no means admit.

The case, upon which so important a conclusion is grounded, is not related with sufficient minuteness. If we understand our author aright, the eye presented the symptoms of cataract combined with glaucoma; it was operated on with the needle through the cornea; the operation was fruitless, and, we presume, followed by inflammation. Is it not much more likely that, in consequence of the inflammation excited by the operation, an effusion took place between the choroid and the retina, than that this effusion, as assumed by Dr. Van der Kolk, preceded the operation, and was the cause of the previous glaucomatous appearance of the eye?

As the matter at present stands, we have little better than a simple averment on the part of Dr. Van der Kolk, that an effusion of coagulable lymph between the choroid and the retina is the cause of glaucoma Glaucomatous eyes have been dissected by Walther, Rosas, Eble, and Mackenzie, and no such effusion of lymph has been noticed by any of these observers.

On the supposition that an effusion of lymph by the choroid is the cause of glaucoma, Dr. Van der Kolk proceeds to explain some of the other symptoms of the disease.

As the sclerotica, he says, possesses a great firmness of texture, not easily admitting of dilatation, it follows, that, by an effusion of lymph between the choroid and retina, the eye must be rendered preternaturally full; whence a feeling of distension, and a gradually increasing hardness of the eye. By the effused substance, the retina will be pressed towards the vitreous humour, whence amaurosis must ultimately ensue. For a time, he thinks the retina will be pressed forwards, so that the focus of the rays of light will no longer fall on it, but behind it, by which means objects will lose their sharp outline, and appear as if in a cloud. At length, not only the retina, but the vitreous humour, the lens, and the iris will be pressed forwards, and the anterior chamber be diminished.

If the lymph, as is always the case at first, is yet clear, the pupil remains black, and a deterioration of vision occurs before any change of colour is to be observed. When the lymph becomes thickened, albuminous and opaque, a change of colour shows itself deep in the bottom of the eye, of a concave form.

An altered or diminished secretion of pigment is also an attendant on chronic inflammation of the choroid; whence the iris becomes pale, and the greenish appearance is augmented. The greenish appearance, however, cannot result from the loss of pigment alone, but depends essentially on the thickened lymph, which assumes, by means of the light entering the eye, a greenish tint.

That a change of the vitreous humour is not the cause of glaucoma, Dr. Van der Kolk concludes, because, first, the greenish tint exhibits a concave and not a convex form, and secondly, the vitreous humour has never shown on dissection a greenish colour, nor a colour which could give rise to such a tint.

We quite agree with our author that the vitreous humour is not the seat of glaucoma; but we cannot say we put much faith in his assertion of the greenish tint exhibiting a concave form. Walther says it is convex. The concave appearance is probably an optical illusion, similar to that by which a cameo sometimes appears as if it were an intaglio. We believe the conclusion, as to the concavity or convexity of the greenish tint in glaucoma, will depend a good deal on the opinion held by the observer regarding its seat. If he regards it as seated at the bottom of the eye, with Dr. Van der Kolk, he will be very apt to see it concave ; if, with Mackenzie, he considers it as seated in the lens, it will seem to him convex.

Dr. Van der Kolk refers to certain occasional changes which the vitreous humour undergoes in colour in glaucoma. It has been found yellowish, blackish gray, reddish or filled with reddish points. These changes of colour he considers as consequences of altered secretion, arising from inflammation communicated to the vessels, already described, of the hyaloid membrane.

Dr. Van der Kolk next criticises the notion of some authors, that the green colour in glaucoma is produced from want of the choroid pigment, and from the action of the amber-coloured lens on the light reflected from the bluish choroidal blood-vessels. He refutes this explanation by the fact, that in old people the choroid is nearly destitute of pigment, and that it is quite so in the albino at all ages, yet there is no green appearance visible in the eye, except in the disease called glaucoma.

Though he is convinced that the greenish colour of the thickened albumen, or coagulable lymph, lying between the retina and the choroid, is the real cause of the glaucomatous appearance, he confesses that a loss of pigment must contribute somewhat to change the blackness of the pupil, and aid in the production of the greenish hue. He refers to the state of two eyes, in which he found the pigment defective : in the one, the choroid, over all the bottom of the eye, was of a light brown colour, but was rather darker towards the anterior part of the eye ; in the other, which had been glaucomatous for six years, the degeneration was much farther advanced, and the effused substance was already more or less organized ; the vitreous humour was partially atrophied ; and the choroid had entirely lost its pigment. In neither of these eyes was there any varicose dilatation of the vessels visible, but he confesses they might have shrunk after death.

That a dilatation of the vessels should occur, is indeed a necessary consequence of inflammation of the choroid, and that such dilatation is a constant phenomenon of glaucoma, Dr. Van der Kolk admits. He thinks that many ophthalmologists have described, under the name of ophthalmitis interna, merely acute choroiditis, while the chronic variety they have designated by the names of glaucoma and cirsophthalmos. His conviction is that the more or less striking signs of glaucoma depend solely on the degree of the exudation between the retina and choroid, which is the essence of the disease, and a consequence of choroiditis.

The effusion varies in quantity in different cases. In some it is small, and retaining its original transparency, it produces no green colour, although all the other symptoms of glaucoma are present. The bottom of the eye, in these cases, presents merely a light cloudy or reddish appearance.

It is an important remark, that, on dissection, if the eye be opened under water, the effusion is not easily detected, unless it be thick and opaque, which may be the cause why it has not been oftener observed.

Dr. Van der Kolk believes that a small degree of effusion frequently occurs, especially in advanced age, between the choroid and retina, and that Jacob's membrane is nothing else than a precipitation of the effused fluid, after death, from the influence of cold, or of the fluid in which the eye is placed, a notion which we consider altogether inadmissible.

Sometimes, he remarks, inflammation of the choroid with effusion has another issue, namely, ossification. Formerly, it was thought that the ossification took place in the retina itself, but later observations have shown that it occurs between the choroid and retina, in consequence of chronic inflammation.

As the vessels of the choroid connect themselves with those of the zonula zinnii, crystalline lens, and hyaloid membrane, the remaining consequences of glaucoma are easily explained. The capsule of the lens receives vessels from the zonula zinnii, and perhaps from the ciliary processes. It is well known, that by long-continued glaucoma, cataract is at last produced. The inflammation of the choroid spreads, according to Dr. Van der Kolk, to the zonuli zinnii, and hence to the capsule; the nutrition of the lens is disturbed, and its transparency destroyed. He seems quite unacquainted with the change in the colour and transparency of the nucleus of the lens, which forms an essential part of the disease called glaucoma, in its earliest stage.

Dr. Van der Kolk gives two figures illustrating the spread of inflammation to the capsule of the lens and lining membrane of the cornea, but as the eye whence these figures were taken was one in which the morbid state of the parts was excited by external injury, they cannot be received as strictly illustrative of glaucoma, although, he says, the eye was glaucomatous and cataractous. In this eye, there was an effusion of fluid between the choroid and retina, vessels were seen spreading from the zonula zinnii to the anterior surface of the capsule, the centre of the capsule was thickened and opaque, and at the same time vessels were found extending from the orbiculus ciliaris and great circumference of the iris to the lining membrane of the cornea.

Our author concludes by repeating, that all the phenomena of glaucoma can be explained without difficulty, from an effusion in consequence of choroiditis, in some cases acute, and in others chronic; and that in the course of the disease, from the connexion of the vessels of the choroid with those of the other parts of the eye, all the affections which are consequences of glaucoma are clearly elucidated. The same original disease, namely, a greater or less degree of choroiditis, has been described, he says, by most authors as different diseases, under the names of ophthalmitis interna, cirsophthalmos, glaucoma, and hydrops oculi. These are only separate issues of the same original affection, namely, choroiditis; the specific character of the inflammation causing in the eye, as in other parts of the body, remarkable differences in the issues.

The plate accompanying Dr. Van der Kolk's paper contains four magnified figures, illustrative of the arrangement of the blood-vessels of the hyaloid membrane, capsule of the lens, and lining membrane of the cornea; and of the effusion of lymph, which he describes between the choroid and retina.

Although we can by no means subscribe to the pathology of glaucoma adopted by the professor, we have perused his paper with much interest, and regard the anatomical part of it as deserving the special attention of our readers.

